# The Role of Hedgehog Signaling in Non-small Cell Lung Cancer: Targeting Tumor Invasion, Therapy Resistance and Novel Therapeutic Strategies

**DOI:** 10.7150/ijbs.123287

**Published:** 2026-01-01

**Authors:** Yu Kang, Hongmei Zheng, Qiuyuan Wen, Songqing Fan

**Affiliations:** 1 Department of Pathology, The Second Xiangya Hospital, Central South University, Changsha, Hunan, 410011, China.; 2 Hunan Clinical Medical Research Center for Cancer Pathogenic Genes Testing and Diagnosis, Changsha, Hunan, 410011, China.

**Keywords:** Hedgehog pathway, epithelial-mesenchymal transition (EMT), non-small cell lung cancer, drug resistance, cancer stem cells (CSCs)

## Abstract

Although essential for normal development and tissue homeostasis, aberrant activation of the Hedgehog (Hh) signaling pathway is implicated in non-small cell lung cancer (NSCLC) progression and treatment resistance. This review details the contribution of Hh signaling to NSCLC, focusing on its promotion of tumor invasion and therapeutic resistance, and establishes a rationale for disrupting this pathway to improve treatment efficacy. Malignant phenotypes in NSCLC are driven by dysregulated Hh pathway activity, often via autocrine or paracrine loops. We specifically assess how Hh pathway activation enables tumor invasion, metastasis, and the development of drug resistance. The review elucidates key resistance mechanisms against diverse therapies—encompassing chemotherapy, targeted therapy and immunotherapy—with a focus on epithelial-mesenchymal transition (EMT), cancer stem cell maintenance, and multidrug resistance (MDR). Therefore, combining Hh pathway inhibitors with standard therapies represents a promising approach for managing treatment-resistant NSCLC.

## 1. Introduction

Globally, lung cancer imposes a severe health burden, with about 2 million new cases and 1.76 million deaths annually 1. Accounting for the majority of cancer-related mortality, non-small cell lung cancer (NSCLC) represents 80-85% of lung cancer cases 2, 3. Overcoming therapeutic resistance and developing effective treatments remain critical challenges. The evolutionarily conserved Hedgehog (Hh) signaling pathway, essential for embryonic patterning and adult tissue homeostasis, is tightly regulated during development, governing core processes such as organogenesis, cell proliferation, differentiation, and survival 4-6. Under normal adult conditions, Hh signaling is largely inactive, contributing to tissue regeneration and homeostasis 7.

Pathological activation of the Hh signaling pathway is associated with disorders such as osteoarthritis, eye deformities, and Gorlin syndrome 8, and drives carcinogenesis in gastrointestinal, skin (e.g., basal cell carcinoma, BCC), and brain (e.g., medulloblastoma) malignancies 9-12. Such dysregulation is observed in approximately 30% of cancers, and approximately one-quarter of tumors depend on Hh signaling for survival 13, 14. The pathway's critical function in cancer development is further evidenced by its pervasive crosstalk with other oncogenic signaling cascades. Thus, key therapeutic strategies involve identifying Hh-responsive biomarkers and developing targeted pathway inhibitors.

## 2. Activation and Dysregulation of the Hh Pathway in Cancer

### 2.1. Activation of the Canonical Hh Signaling Pathway

Activation of the canonical Hh signaling pathway is initiated by the binding of Hh ligands to Patched (PTCH) receptors. Three distinct Hh ligands are expressed in mammals: sonic hedgehog (SHH), Indian hedgehog (IHH), and desert hedgehog (DHH). These ligands interact with two PTCH receptors, PTCH1 and PTCH2, with PTCH1 functioning as the principal Hh receptor 15. The intercellular transport of lipid-modified SHH, which has low solubility, relies on its association with specific carrier proteins like signal peptide-CUB-EGF-like domain-containing protein 2 (SCUBE2) 16. Growth arrest-specific 1 (GAS1) binds doubly lipidated SHH presented by the extracellular carrier SCUBE2. GAS1 then directly interacts with PTCH1, which transfers SHH to form an SHH-PTCH complex 17, 18. Following endocytosis and lysosomal degradation of this complex, the suppression of smoothened (SMO) is relieved 18. In the absence of Hh signaling, PTCH inhibits SMO by blocking access of endogenous sterol ligands to the binding sites on SMO 19, 20. Activated SMO translocates to the primary cilium tip, where phosphorylation by PKA and casein kinase 1 alpha (CK1α) prevents its degradation, thereby stabilizing the protein. SMO subsequently initiates a phosphorylation cascade that inhibits suppressor of Fused (SUFU), leading to the activation of the GLI family zinc finger (GLI) transcription factors. Primary cilia, which are microtubule-based signaling organelles, are essential for this pathway 21. The GLI family comprises three members: GLI1 and GLI2 function primarily as transcriptional activators, whereas GLI3 acts mainly as a repressor 14, 22. SUFU sequesters GLI transcription factors in the cytoplasm within a multiprotein complex tethered to microtubules, thereby preventing their activation 23. This protein also modulates the entry of GLI into the nucleus and its transcriptional function 24. Upon activation by SMO, GLI proteins are released from SUFU, processed into their transcriptionally active state, and translocate to the nucleus to drive target gene expression. GLI drives cellular responses like proliferation and survival by upregulating Cyclin D and anti-apoptotic factors such as B-cell lymphoma 2 (Bcl-2) 25-27. Furthermore, GLI regulates the pathway itself by inducing the expression of key components including PTCH and Hedgehog-interacting protein (HHIP), creating feedback loops that control pathway intensity. HHIP, a membrane glycoprotein, inhibits the pathway by binding Hh ligands, leading to the ligands' internalization and degradation 28. Notably, HHIP expression is itself upregulated by Hh pathway activation through a negative feedback loop, thereby self-limiting signaling. Reduced HHIP expression in lung cancer has been correlated with inhibited tumor growth, indicating its context-dependent role in oncogenesis 29.

### 2.2. Aberrant Activation of the Hh Pathway

Oncogenic Hh pathway activation occurs through distinct mechanisms, which can be classified as: (i) ligand-independent pathways (e.g., mutations in the *PTCH1* or *SMO* genes) and (ii) ligand-dependent autocrine/paracrine signaling. Both modes promote tumor growth and metastatic dissemination. Constitutive, ligand-independent activation of the Hh pathway arises from genetic defects such as loss-of-function *PTCH* mutations, gain-of-function *SMO* modifications, or *GLI1* amplification. This dysregulated activation is a common feature in tumors like medulloblastoma, neuroblastoma, and rhabdomyosarcoma 30, 31. The ligand-dependent activation mechanism involves Hh ligands—produced by tumor cells or the surrounding microenvironment—that promote tumor growth. This form of activation, frequently observed in primary NSCLC, responds to Hh pathway inhibitors, including SMO antagonists 32. Additionally, paracrine Hh signaling remodels the tumor microenvironment, facilitating tumor progression in malignancies such as gastrointestinal, pancreatic, prostate, and lung cancers 33-37.

### 2.3. Crosstalk with Other Signaling Pathways

**Figure 1:**
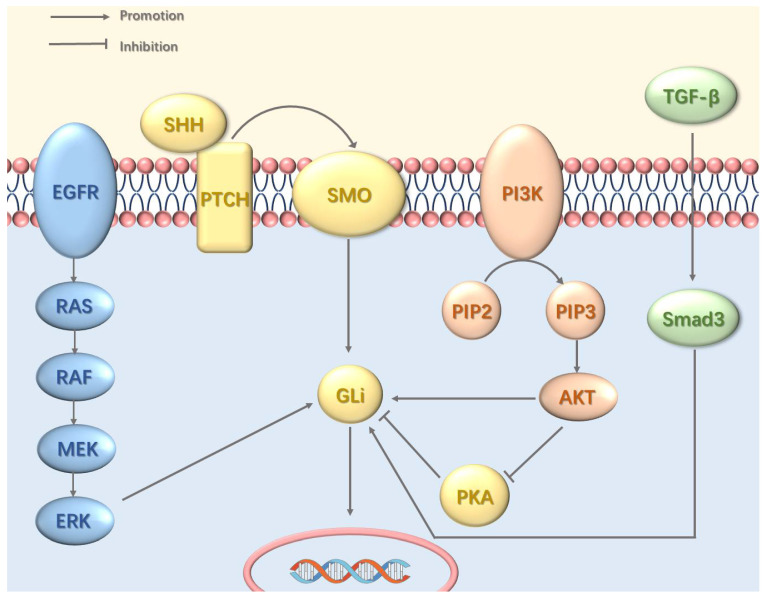
Crosstalk between the Hh Pathway and Other Signaling Pathways. The Hh pathway can be persistently activated through crosstalk with other signaling pathways, including EGFR, PI3K-Akt, and TGF-β. EGFR signaling induces Gli activation via the MAPK cascade. The PI3K-Akt pathway directly activates Gli transcription factors and concurrently enhances Hh signaling by suppressing PKA, a key negative regulator of the pathway. TGF-β further promotes the transcriptional upregulation of GLI.

Interactions between the Hedgehog pathway and other oncogenic signaling cascades contribute to sustained GLI activity and tumorigenesis (Figure 1). This crosstalk involves multiple mechanisms including the epidermal growth factor receptor—mitogen-activated protein kinase (EGFR-MAPK) pathway, which mediates GLI phosphorylation via MAPK 38; the phosphoinositide 3-kinase (PI3K)-protein kinase B (AKT) pathway, which promotes GLI nuclear entry and inhibits its protein kinase A (PKA)-dependent inactivation 39; and the transforming growth factor-beta (TGF-β)-SMAD pathway, which drives transcriptional upregulation of GLI expression 40.

## 3. Hh Pathway in Lung Cancer

Studies have established that the Hh pathway is frequently activated in NSCLC and correlates with the histological characteristics and prognosis of tumors 41-43. Elevated SHH expression, which is associated with poor tumor differentiation 44, is also observed in drug-resistant NSCLC cell lines. Huang *et al.* reported the activation of Hh signaling across distinct NSCLC subtypes, with the highest levels observed in lung squamous cell carcinoma (LSCC) 45, 46.

## 4. The Role of the Hh Pathway in Lung Cancer Progression

### 4.1 Promoting Lung Cancer Cell Proliferation

The Hh pathway is typically maintained at low activity levels in adult tissues; however, its aberrant activation has been shown to promote tumor growth and progression 6. Multiple studies have demonstrated that inhibition of the Hh pathway suppresses cellular proliferation in lung cancer cells 47, 48. For instance, knockdown of GLI in lung squamous cell carcinoma significantly inhibits cancer cell proliferation and induces apoptosis 46. Furthermore, as one of the key pathways regulating cancer stem cells (CSCs), the Hh pathway plays a central role in maintaining stemness and self-renewal in CSCs and this sustained CSC population can further enhance the proliferation of lung cancer cells 49.

### 4.2 Mediating Cancer Cell Invasion and Metastasis

**Figure 2:**
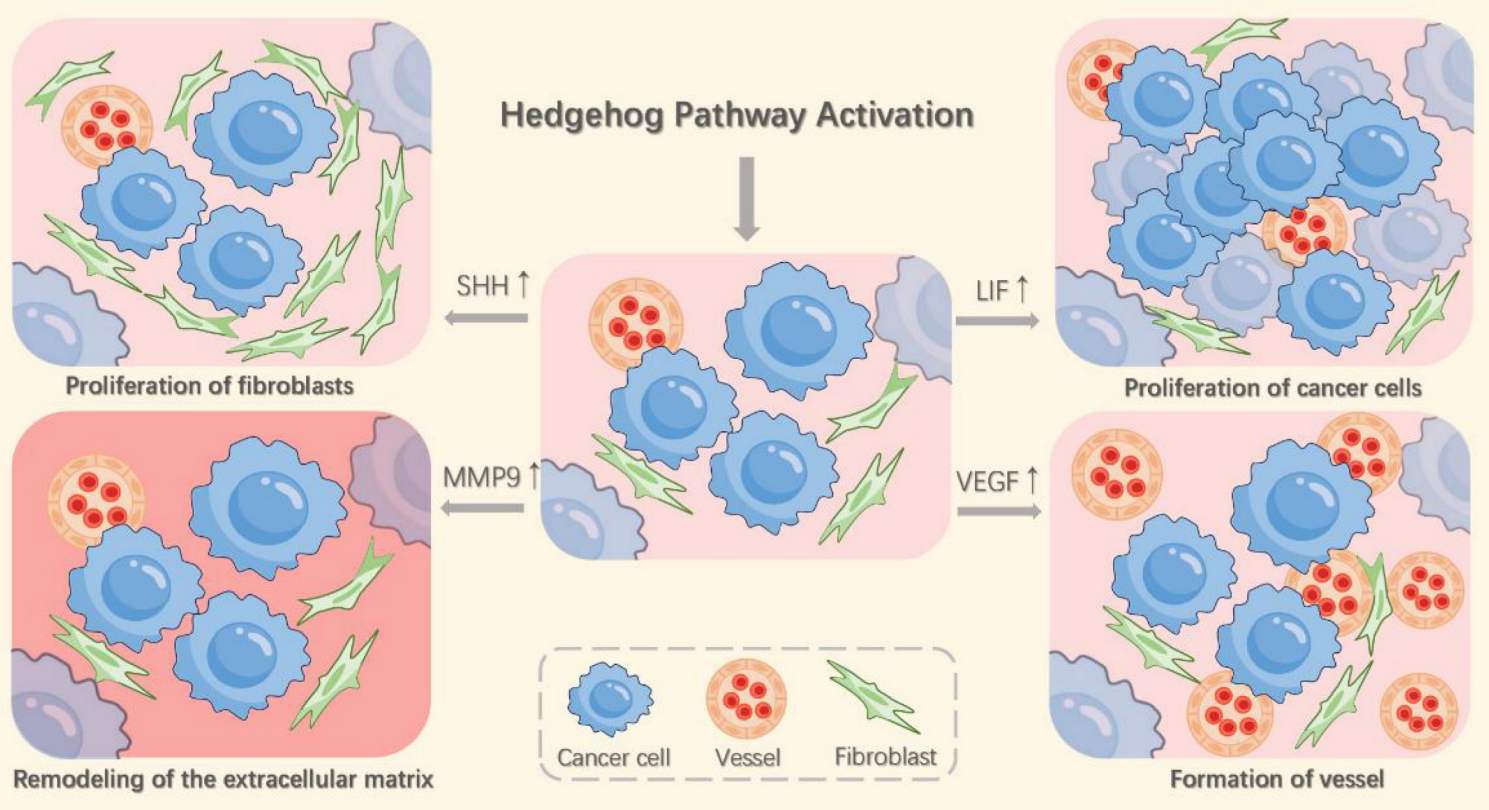
Hh pathway activation remodels the tumor microenvironment by stimulating angiogenesis, lymphangiogenesis, extracellular matrix reorganization, and peritumoral fibroblast expansion. These fibroblasts secrete factors including LIF, which enhances tumor cell proliferation, and VEGF, which drives angiogenesis.

The Hh pathway, activated in lung fibroblasts by SHH secreted from tumor cells in NSCLC, plays a critical role in enhancing cancer cell invasiveness and metastasis (Figure 2) 48. These activated fibroblasts respond by secreting mediators, including vascular endothelial growth factor (VEGF) and leukemia inhibitory factor (LIF). VEGF promotes tumor angiogenesis, while LIF increases the proliferation and metastatic potential of NSCLC cells 50, 51. Additionally, Hh pathway stimulation induces fibroblast proliferation and invasion, forming "tunnels" that facilitate cancer cell spread 52. It also triggers fibroblasts to synthesize and deposit collagen and to upregulate matrix metalloproteinase-9 (MMP9), thereby promoting extracellular matrix (ECM) remodeling 48. The degradation of ECM components like collagen and elastin by MMP9 highlights the importance of fibroblast-driven processes in tumor cell migration and invasion 53.

The Hh signaling pathway critically drives tumor progression by stimulating angiogenesis and lymphangiogenesis, processes essential for tumor expansion and lymph node metastasis 54. Specifically, SHH signaling promotes blood vessel formation in pathological settings like myocardial infarction and stroke 55. However, inhibiting the SHH/GLI1 pathway pharmacologically can counteract tumor-related angiogenesis. In NSCLC, activation of this pathway spurs endothelial cell proliferation and migration, recruits pericytes to new vessels, and consequently fosters the formation and stabilization of vascular networks 56, 57. Suppressing Hh pathway signaling inhibits angiogenesis and elevates reactive oxygen species (ROS) levels 58. Although SHH activates vascular endothelial growth factor receptor 2 (VEGFR2) phosphorylation, the mechanistic link between the SHH/GLI1 pathway and VEGF/VEGFR2 signaling remains unclear and requires further investigation. Inhibiting the SHH/GLI1 pathway suppresses tumor growth, primarily through reduced angiogenesis. Furthermore, Hh signaling enhances lymphatic vessel endothelial hyaluronan receptor 1 (LYVE-1) expression, indicating a role in lymphangiogenesis. Given the strong correlation between tumor lymphangiogenesis and regional lymph node metastasis 59, 60, Hh signaling is likely to promote lung cancer metastasis by stimulating lymphangiogenesis. Overactivation of this pathway could therefore serve as a potential biomarker for predicting lymph node metastasis in NSCLC.

### 4.3 Fostering the Self-Renewal Ability of Cancer Stem Cells

A subpopulation known as CSCs, which exhibit stem-like properties, drives tumor relapse, metastasis, and therapy resistance. CSCs are thought to originate from either normal stem cells or progenitor cells. In mouse models of lung cancer, lung CSCs are located within unique niches that support their stemness, adaptability, immune evasion, and metastatic potential. The Wnt/β-catenin, Notch, and Hh pathways are fundamental in regulating stemness 61, 62. Notably, embryonic transcription factors, including SRY-box transcription factor 2 (SOX2), octamer-binding transcription factor 4 (OCT4), and Nanog homeobox (NANOG), are vital for preserving the self-renewal capacity and undifferentiated state of CSCs in various cancers.

**Figure 3:**
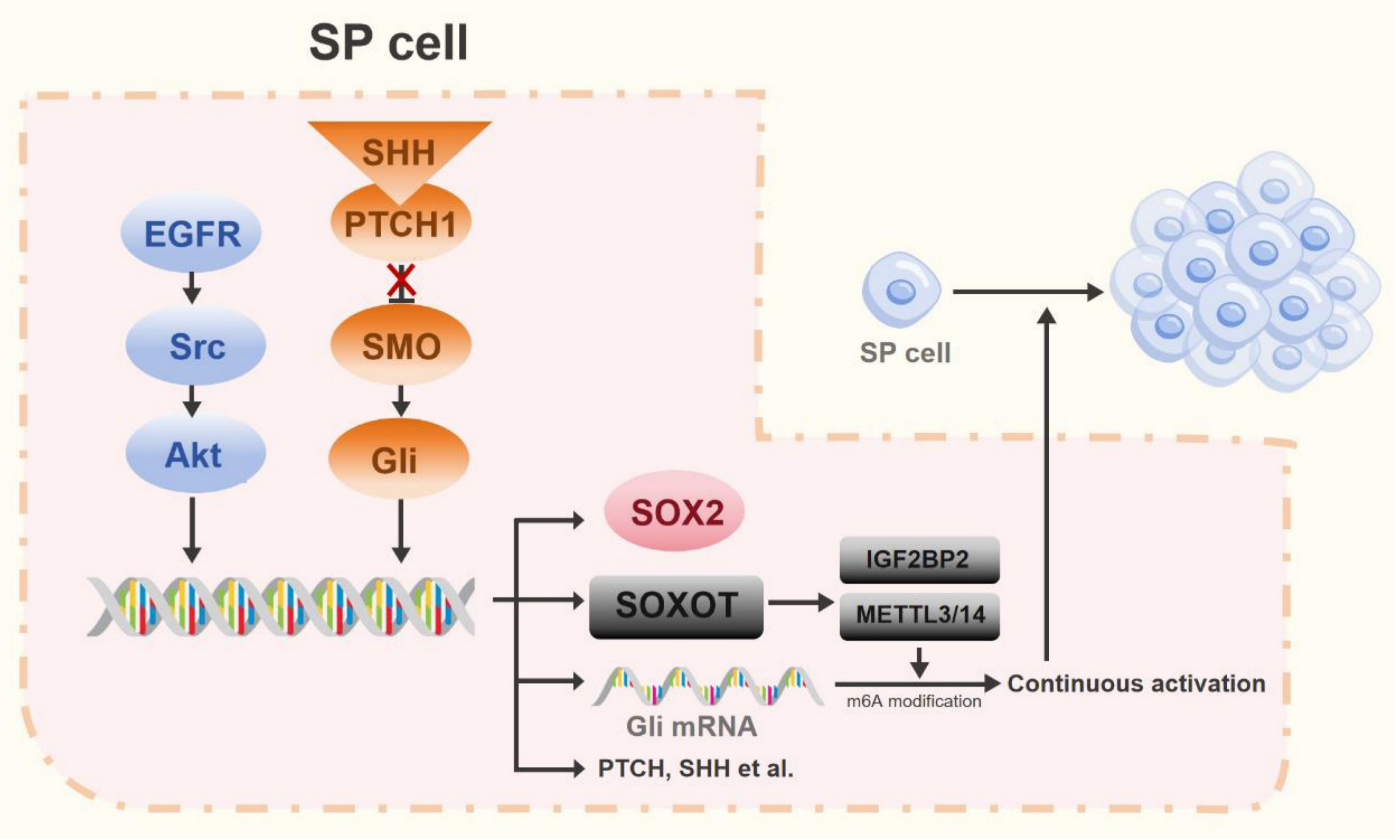
The Hh pathway in stem cell-like side population (SP) cells. In SP cells, the EGFR-Src-Akt pathway and the Hh pathway cooperatively regulate stemness by inducing SOX2 expression. Furthermore, activation of the Hh pathway promotes expression of the long non-coding RNA *SOX2OT*.* SOX2OT* facilitates m^6^A methylation of *Gli* transcription factors via the METTL3/14-IGF2BP2 complex, establishing a positive feedback loop that amplifies Hh signaling.

Experimental studies in rat models of lung cancer have established that Hh pathway activation correlates with elevated expression of CSC markers, including OCT4, SOX2, cluster of differentiation 133 (CD133), ATP-binding cassette subfamily G member 2 (ABCG2), and NANOG 49, 63. In NSCLC, the stem cell-like side-population (SP) exhibits key stem cell properties that are primarily regulated by the transcription factor SOX2 64. SOX2, a critical transcription factor, governs self-renewal, pluripotency, and reprogramming in undifferentiated embryonic stem cells 65. Earlier studies demonstrated that impaired Hh signaling reduces SOX2 levels, consequently weakening the self-renewal capacity and vasculogenic mimicry of SP cells. The transcription factor GLI1 cooperates with the EGFR pathway to stimulate SOX2 expression, thereby influencing SP cell stemness and function 64, 66. Recent work uncovered a non-canonical mechanism for GLI1 activation that bypasses the SHH/PTCH/SMO pathway. This involves GLI1-mediated upregulation of methyltransferase-like 3/14 (METTL3/14) and insulin-like growth factor 2 mRNA-binding protein 2 (IGF2BP2) through modulation of the SOX2 overlapping transcript (SOX2OT). These enzymes then promote N6-methyladenosine (m6A) RNA methylation to facilitate GLI1 activation 67. GLI1, along with its downstream effectors, promotes proliferation in both lung cancer cells and CSCs. Furthermore, EGFR-Src family kinase (SRC)-AKT signaling triggers transcriptional upregulation of SOX2 in SP cells, thereby promoting their self-renewal 64. Collectively, these findings reveal a synergistic interaction between EGFR and Hh pathways, which converge to regulate SOX2 transcription, thereby jointly controlling the stem cell-like characteristics of SP cells in NSCLC (Figure 3).

Targeting the Hh pathway shows significant preclinical promise for combating therapy-resistant lung CSCs. Agents such as sulforaphane inhibit CSC self-renewal by disrupting Hh activity and downregulating polyhomeotic homolog 3 (PHC3), while PHC3 interacts with the Hh pathway to maintain the survival of tumor stem cells 68. Hh inhibitors combined with radiotherapy reduce CSC populations and EMT markers, and glucocorticoids can suppress CSCs via Hh/SOX2 inhibition 63, 69. Collectively, these findings confirm that Hh signaling governs CSCs' gene expression profiles and maintenance mechanisms (self-renewal, proliferation, and metastasis). Given that EMT underpins CSC niche formation and CSCs drive resistance to chemotherapy and radiotherapy, Hh pathway inhibition offers a strategy to deplete CSCs and enhance treatment sensitivity. The potent anti-CSCs efficacy of Hh-targeting agents in lung cancer models underscores their therapeutic potential for NSCLC 70.

### 4.4 The Hh Pathway Promotes Therapy Resistance by Inducing EMT in Cancer Cells

EMT, induced by factors including TGF-β, fibroblast growth factor (FGF), and epidermal growth factor (EGF), contributes to resistance against various cancer therapies 71. Defined by the loss of epithelial markers (e.g., E-cadherin, ZO-1) and gain of mesenchymal markers (e.g., N-cadherin, vimentin) 72, EMT in lung cancer is significantly driven by Hh pathway activation. This activation suppresses epithelial traits and enhances mesenchymal characteristics, thereby promoting therapy resistance 73. The Hh pathway inhibition (e.g., via GLI1 suppression) reverses EMT, restores epithelial marker expression, and reduces cancer cell migration and invasion, demonstrating the plasticity of this process 74, 75. Thus, Hh-mediated EMT is a key contributor to lung cancer therapy resistance, and targeting this pathway offers a promising strategy to restore treatment sensitivity.

## 5. The Role of the Hh Pathway in Lung Cancer Treatment

**Figure 4:**
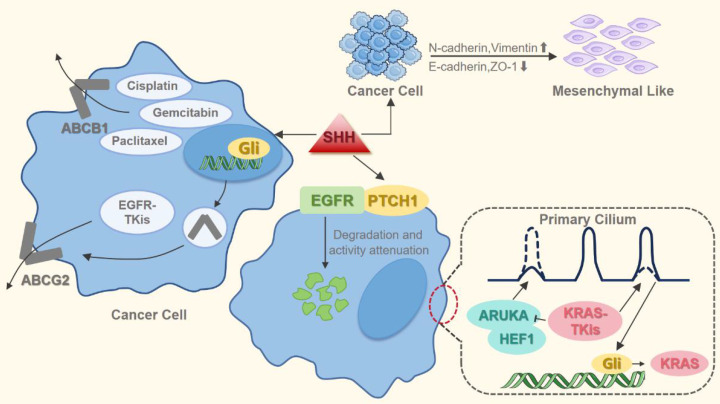
Hh pathway activation induces drug resistance in lung cancer cells. This occurs primarily through EMT mediation and MDR induction. Specifically, Hh signaling upregulates MDR efflux transporters (e.g., ABCG2 and ABCB1), enhancing drug extrusion and reducing intracellular chemotherapeutic concentrations. Furthermore, the pathway stimulates KRAS expression, which confers resistance to KRAS-targeted inhibitors.

In lung cancer, aberrant activation of the Hh signaling pathway significantly contributes to multidrug resistance (MDR) that extends beyond its role in tumor progression (Figure 4). Specifically, Hh overactivation in NSCLC drives resistance to treatments like epidermal growth factor receptor-tyrosine kinase inhibitors (EGFR-TKIs), platinum-based drugs, and other chemotherapeutic agents. The pathway's core effector, GLI1, directly influences EGFR-TKI sensitivity 76. Mechanistically, Hh pathway activation induces EMT in EGFR-mutant NSCLC cells, leading to EGFR-TKI resistance that can be reversed by pharmacological inhibition of the pathway 74. Furthermore, Hh inhibition reduces the expression of MDR transporters such as ATP-binding cassette subfamily B member 1 (ABCB1) and ABCG2, enhancing tumor cell chemosensitivity 77, 78. Collectively, these studies establish dysregulated Hh signaling as a major driver of MDR in lung cancer cells.

Research confirms that acquired resistance to EGFR-TKIs is associated with EMT and an enhanced CSC phenotype 62, 79. The Hh signaling pathway not only plays a critical role in maintaining the stemness of lung cancer stem cells but also serves as one of the key drivers of EMT. Furthermore, the Hh pathway promotes PTCH1-mediated ubiquitination and proteasomal degradation of EGFR, reducing its activity in NSCLC cells 80. Therefore, inhibiting Hh pathway could improve EGFR-TKI efficacy in NSCLC. However, Hh inhibition alone is often insufficient to overcome resistance; effective suppression of downstream EGFR effectors requires concurrently targeting both EGFR and the Hh pathway. Experimental evidence has demonstrated that the combination of itraconazole and osimertinib significantly suppressed the proliferation of osimertinib-resistant cells and inhibited the growth of osimertinib-resistant tumors 81. These findings provide a preclinical rationale for combining Hh inhibitors with EGFR-TKIs, which may be a promising strategy against EGFR-TKI resistance in NSCLC. However, rigorous clinical validation of its efficacy and safety remains crucial.

The Hh pathway is a critical modulator of chemotherapy resistance in NSCLC, impacting response to drugs such as cisplatin and paclitaxel 68, 73. Multiple Hh pathway-related genes are upregulated in cisplatin-resistant cells compared to cisplatin-sensitive cells 82. The Hh pathway's activation confers resistance primarily by enhancing MDR via ATP-binding cassette (ABC) transporter-dependent drug efflux and inducing protective mechanisms such as BCL2-associated X protein (BAX) phosphorylation, particularly against paclitaxel 83-86. Notably, paclitaxel treatment itself can competitively activate Hh signaling. This activation antagonizes chemotherapy-induced cell death by inhibiting mitochondrial damage, ROS accumulation (essential for paclitaxel-induced apoptosis), and mitotic catastrophe 83, 87. Synergistically blocking Hh signaling (e.g., reducing ABCB1 levels) sensitizes NSCLC cells to chemotherapy, highlighting the therapeutic potential of combined Hh inhibitor and chemotherapy regimens 84, 85. Although prior research identifies Kirsten rat sarcoma viral oncogene homolog (KRAS) re-expression as a major driver of acquired resistance to KRAS inhibitors, its molecular basis was poorly understood 88. KRAS-mutant cancers exhibit elevated SHH signaling 89. Work by Lee *et al.* shows that KRAS inhibitors trigger primary cilia formation and downregulate Aurora kinase A (AURKA) 90. AURKA serves as a key cilium disassembly factor, whereas Hh signal transduction requires the formation of primary cilia 91, 92. The downregulation of AURKA induces Hh signaling, leading to the transcription factor GLI1 directly binds to the KRAS promoter, promoting KRAS transcription and protein expression, ultimately leading to drug resistance 90. Therefore, the Hh signaling pathway plays a key role in the development of resistance to KRAS inhibitors in NSCLC. This suggests that combined inhibition of the Hh and KRAS pathways may overcome acquired resistance to KRAS inhibitors.

**Figure 5:**
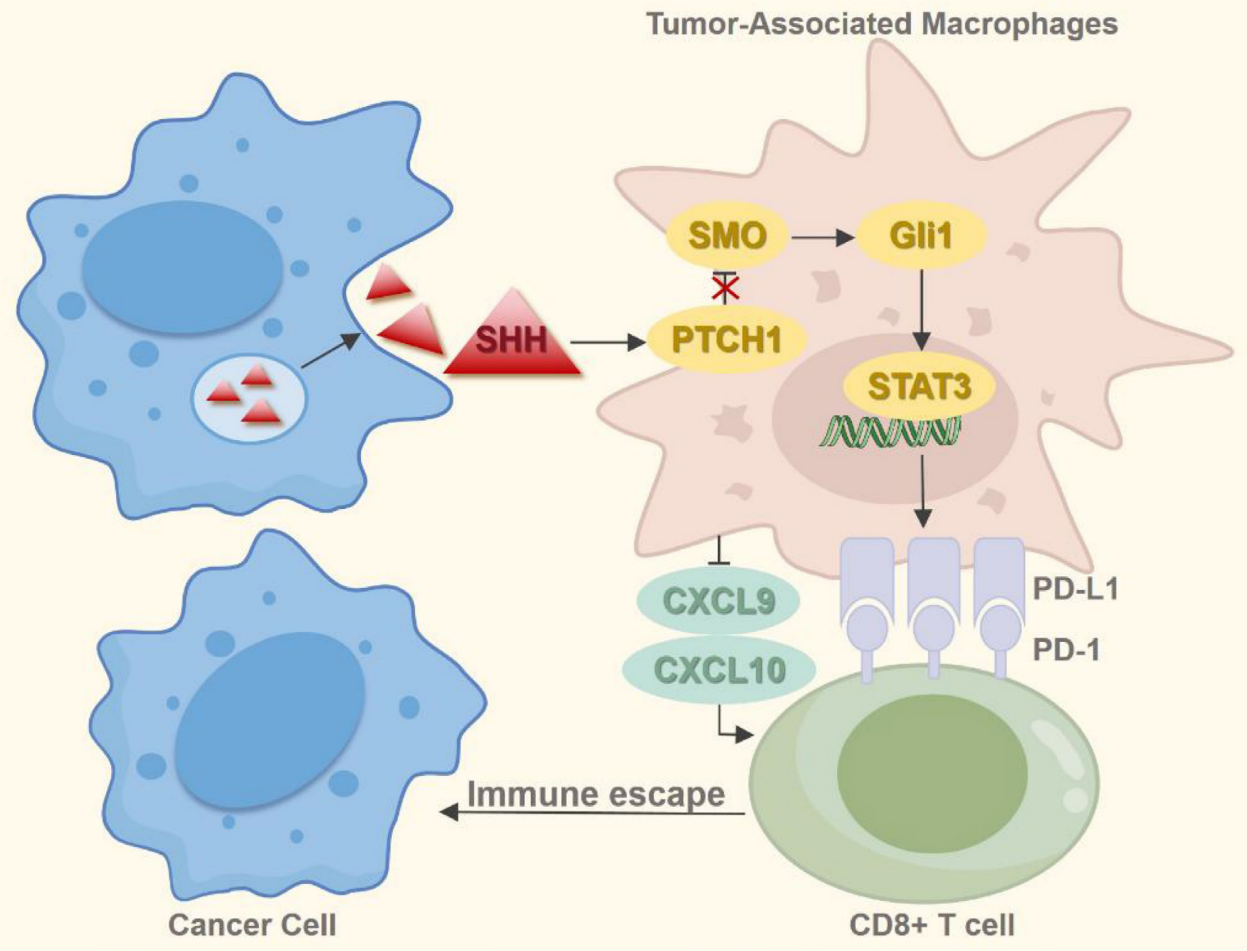
The Hh pathway's role in immune microenvironment. Tumor cells secrete SHH, which acts on TAMs, activating the SHH-Gli1-STAT3 signaling cascade and upregulating PD-L1 expression on TAMs. This process impairs CD8⁺ T cell function. Additionally, TAMs inhibit CD8⁺ T cell infiltration into the TME by suppressing the production of chemokines CXCL9 and CXCL10, thereby facilitating tumor immune escape and promoting progression.

Research implicates the Hh signaling pathway in both the development and activation of immune responses 93. Inhibition of this pathway reprograms the dysregulated immune microenvironment in breast cancer, contrasting with BCC where Hh pathway activation promotes an immunosuppressive state 94, 95. Experiments by Petty *et al.* demonstrated that tumor-derived SHH directly induces the polarization of tumor-associated macrophages (TAMs) 96. These M2-polarized TAMs enhance tumor angiogenesis, foster cancer stem cell-like properties, and contribute to chemotherapy resistance 97. Furthermore, they impair CD8^+^ T-cell recruitment into the tumor microenvironment (TME) by suppressing the production of chemokines C-X-C motif chemokine ligand 9 and 10 (CXCL9/CXCL10), consequently facilitating tumor progression 96. TAMs express programmed cell death-ligand 1 (PD-L1), and stromal PD-L1 within tumors suppresses intratumoral CD8^+^ T-cell function. Notably, Hh signaling regulates TAM PD-L1 expression via SHH-mediated GLI1 activation, which subsequently stimulates the signal transducer and activator of transcription 3 (STAT3) to upregulate PD-L1. This process results in CD8^+^ T-cell dysfunction and immune evasion 98. In NSCLC, PD-L1 is more frequently expressed on immune cells than on tumor cells. Evidence therefore suggests that the SHH-GLI1-STAT3 axis may contribute to immune suppression and resistance to immune checkpoint inhibitors (ICIs) through mechanisms similar to those documented in other malignancies, though further experimental confirmation is required (Figure 5). Activation of the Hh pathway during ICI therapy correlates with poorer outcomes, suggesting Hh pathway markers could serve as prognostic biomarkers for NSCLC patients 99. Comutation in the Hh and the spliceosome pathway is a recognized predictors of elevated tumor mutational burden (TMB) and neoantigen load (NAL), which are potential indicators of response to immunotherapy 100. Modulating the NSCLC immune microenvironment by targeting the Hh pathway represents a promising therapeutic approach. Given the pathway's role in driving immune evasion and ICI resistance, combining Hh inhibitors with ICIs offers a promising strategy to overcome therapeutic resistance and enhance efficacy. Nevertheless, resistance to SMO inhibitors consistently emerges in the clinic, possibly due to activation of GLI transcription factors through alternative signaling or compensatory pathways. Consequently, the advancement of therapeutic strategies requires identifying novel biomarkers for precise patient stratification (identifying Hh inhibitor-responsive subsets) and real-time monitoring of treatment effectiveness.

## 6. Discussion

Clinical evidence consistently indicates that aberrant Hh pathway activation significantly influences lung cancer pathogenesis, driving tumor growth, progression, therapy resistance, and ultimately poor outcomes. Studies link elevated GLI1 expression to lower survival rates 67, and associate SHH overexpression with a poor prognosis in small cell lung cancer (SCLC) 101. Therefore, the development of novel Hh pathway biomarkers holds clinical significance for predicting outcomes in lung cancer patients. Targeting this pathway, which regulates EMT, CSC properties, and multidrug resistance, holds therapeutic potential. The cross-talk of the Hh pathway with various oncogenic signaling cascades is outlined in Table 1. Therapeutically, the principal molecular target for approved Hh inhibitors is the SMO receptor. Clinically used SMO antagonists include vismodegib and sonidegib, indicated for BCC, and glasdegib, used in acute myeloid leukemia (AML) 102-104. Their mechanism of action involves binding to SMO to impede downstream signaling. A significant limitation of these drugs is the frequent development of resistance, commonly arising from SMO mutations or activation of alternative, non-canonical pathways. In preclinical models, a contrasting approach—directly inhibiting the GLI transcription factors (e.g., GLI2 with GANT61)—has shown substantial anti-tumor activity 46. This contrasts with the limited efficacy of SMO inhibitors like vismodegib in lung adenocarcinoma, where they fail to inhibit GLI2 105. This mechanistic shortcoming aligns with the overall lack of success in clinical trials testing SMO inhibitor monotherapy for NSCLC. Illustratively, a window-of-opportunity study using high-dose itraconazole reported reductions in tumor volume and pro-angiogenic factors but no corresponding downregulation of Hh target genes like *GLI1* and *PTCH* 106, implying that the observed effects may be off-target or that Hh signaling was not sufficiently suppressed. The collective lack of positive clinical data suggests that NSCLC tumorigenesis is not primarily driven by the Hh pathway but is a consequence of complex, interconnected signaling networks. Thus, a critical future direction is to define the mechanisms underlying resistance to Hh inhibition. Given the constraints of Smo-targeted drugs, a promising avenue for future therapy is the development of agents capable of directly inhibiting GLI transcription factors, particularly GLI2.

**Figure 6:**
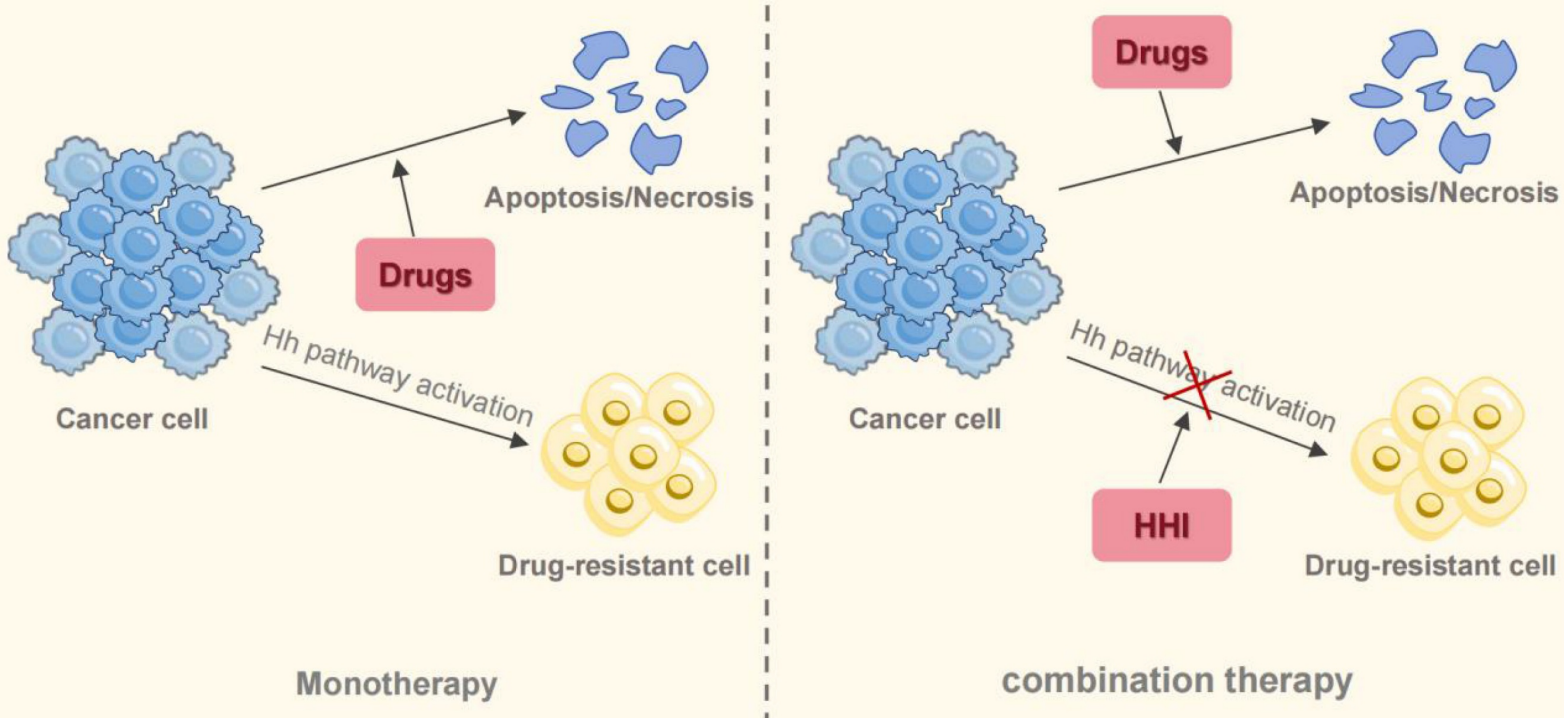
Combination therapy to overcome drug resistance in NSCLC. Monotherapy for NSCLC inevitably leads to drug resistance. The activation of the Hh signaling pathway contributes to this resistance through multiple mechanisms. Combining targeted agents or chemotherapeutic drugs with HHI resensitizes tumors to standard treatments, offering a promising therapeutic strategy.

Substantial preclinical data provide a compelling basis for combining Hh inhibitors (HHIs) with other agents, a strategy that appears more promising than HHI monotherapy for countering resistance to EGFR-TKIs and chemotherapy, as well as for altering the immune landscape of tumors (Figure 6). Emerging clinical data from phase II trials support this premise. One such study (NCT03664115), a randomized phase II trial, reported that combining itraconazole with platinum-based chemotherapy led to significantly better objective response rates (ORR) and progression-free survival (PFS) than chemotherapy by itself 107. The exploration of this strategy continues, with additional clinical trials (e.g., NCT04007744, NCT06616623) now evaluating HHI and immunotherapy combinations. Therefore, integrating HHIs into existing treatment protocols represents a viable path to address therapeutic resistance in NSCLC. However, the definitive clinical benefit of this approach is not yet established, pending positive results from large-scale phase III trials.

## Figures and Tables

**Table 1:** The cross-talk of the Hh pathway with various oncogenic signaling cascades

Pathway	Interaction with Hedgehog Pathway
EGFR-RAS-RAF-MEK-ERK	Mediates phosphorylation of GLI
PI3K-AKT-PKA	Induces upregulation of GLI expression
TGF-β-SMAD3	Induces upregulation of GLI expression
EGFR-SRC-AKT-SOX	Cooperates with Hh pathway in regulating stem-like properties of SP cells
SHH-GLI1-STAT3	Mediates immune escape
AURKA-Hh-GLI-KRAS	Activates Hh signaling leading to KRAS re-expression

## Abbreviations

ABC: transporter ATP-binding cassette transporter
ABCB1: ATP-binding cassette subfamily B member 1
ABCG2: ATP-binding cassette subfamily G member 2
AKT: protein kinase B
AML: acute myeloid leukemia
AURKA: aurora kinase A
BAX: Bcl2-associated x protein
BCC: basal cell carcinoma
Bcl-2: B-cell lymphoma 2
CD133: cluster of differentiation 133
CK1α: casein kinase 1 alpha
CSCs: cancer stem cells
CXCL9/10: C-X-C motif chemokine ligand 9/10
DHH: desert hedgehog
E-cadherin: epithelial-cadherin
ECM: extracellular matrix
EGF: epidermal growth factor
EGFR: epidermal growth factor receptor
EGFR-TKIs: epidermal growth factor receptor-tyrosine kinase inhibitors
EMT: epithelial-mesenchymal transition
FGF: fibroblast growth factor
GAS1: growth arrest-specific 1
GLI: family zinc finger
GM-CSF: granulocyte-macrophage colony-stimulating factor
Hh: Hedgehog
HHIP: Hedgehog-interacting protein
HHI: Hedgehog pathway inhibitors
ICIs: immune checkpoint inhibitors
IGF2BP2: insulin-like growth factor 2 mRNA-binding protein 2
IHH: Indian hedgehog
IL-1β: interleukin-1 beta
KRAS: kirsten rat sarcoma viral oncogene homolog
LIF: leukemia inhibitory factor
LSCC: lung squamous cell carcinoma
LYVE-1: lymphatic vessel endothelial hyaluronan receptor 1
MAPK: mitogen-activated protein kinase
MDR: multidrug resistance
METTL3/14: methyltransferase-like 3/14
MMP9: matrix metalloproteinase-9
m6A: N6-methyladenosine
NAL: neoantigen load
NANOG: Nanog homeobox
N-cadherin: neural-cadherin
NSCLC: non-small cell lung cancer
OCT4: octamer-binding transcription factor 4
ORR: objective response rate
PD-L1: programmed cell death-ligand 1
PFS: progression-free survival
PHC3: polyhomeotic homolog 3
PI3K: phosphoinositide 3-kinase
PKA: protein kinase A
PTCH: patched
ROS: reactive oxygen species
SCLC: small cell lung cancer
SCUBE2: signal peptide-CUB-EGF-like domain-containing protein 2
SHH: sonic hedgehog
Smad: sma and mad-related proteins
SMO: smoothened
Sox2: SRY-box transcription factor 2
SOX2OT: SOX2 overlapping transcript
SP: side population
SRC: src family kinase
STAT3: signal transducer and activator of transcription 3
SUFU: suppressor of fused
TAMs: tumor-associated macrophages
TGF-β: transforming growth factor-beta
TMB: tumor mutational burden
TME: tumor microenvironment
VEGF: vascular endothelial growth factor
VEGFR2: vascular endothelial growth factor receptor 2
ZO-1: zonula occludens-1

## References

[1] Thai AA, Solomon BJ, Sequist LV, Gainor JF, Heist RS (2021). Lung cancer. Lancet (London, England).

[2] Wang M, Herbst RS, Boshoff C (2021). Toward personalized treatment approaches for non-small-cell lung cancer. Nature medicine.

[3] Zou Y, Zheng H, Ning Y, Yang Y, Wen Q, Fan S (2023). New insights into the important roles of phase seperation in the targeted therapy of lung cancer. Cell & bioscience.

[5] Petrova R, Joyner AL (2014). Roles for Hedgehog signaling in adult organ homeostasis and repair. Development (Cambridge, England).

[6] Liu H, Zhou D, Ou Y, Chen S, Long Y, Yuan T (2025). Multiple signaling pathways in the frontiers of lung cancer progression. Frontiers in immunology.

[7] Amakye D, Jagani Z, Dorsch M (2013). Unraveling the therapeutic potential of the Hedgehog pathway in cancer. Nature medicine.

[8] Onodera S, Azuma T (2023). Hedgehog-Related Mutation Causes Bone Malformations with or without Hereditary Gene Mutations. International journal of molecular sciences.

[9] Xie J, Murone M, Luoh SM, Ryan A, Gu Q, Zhang C (1998). Activating Smoothened mutations in sporadic basal-cell carcinoma. Nature.

[10] Bossi P, Ascierto PA, Basset-Seguin N, Dreno B, Dummer R, Hauschild A (2023). Long-term strategies for management of advanced basal cell carcinoma with hedgehog inhibitors. Critical reviews in oncology/hematology.

[11] Zhang J, Fan J, Zeng X, Nie M, Luan J, Wang Y (2021). Hedgehog signaling in gastrointestinal carcinogenesis and the gastrointestinal tumor microenvironment. Acta pharmaceutica Sinica B.

[12] Qu M, He Q, Luo J, Shen T, Gao R, Xu Y (2023). Sonic hedgehog signaling: Alternative splicing and pathogenic role in medulloblastoma. Genes & diseases.

[13] Murone M, Rosenthal A, de Sauvage FJ (1999). Hedgehog signal transduction: from flies to vertebrates. Experimental cell research.

[14] Lum L, Beachy PA (2004). The Hedgehog response network: sensors, switches, and routers. Science (New York, NY).

[15] Varjosalo M, Taipale J (2008). Hedgehog: functions and mechanisms. Genes & development.

[16] Tukachinsky H, Kuzmickas RP, Jao CY, Liu J, Salic A (2012). Dispatched and scube mediate the efficient secretion of the cholesterol-modified hedgehog ligand. Cell reports.

[17] Huang P, Wierbowski BM, Lian T, Chan C, García-Linares S, Jiang J (2022). Structural basis for catalyzed assembly of the Sonic hedgehog-Patched1 signaling complex. Developmental cell.

[18] Torroja C, Gorfinkiel N, Guerrero I (2004). Patched controls the Hedgehog gradient by endocytosis in a dynamin-dependent manner, but this internalization does not play a major role in signal transduction. Development (Cambridge, England).

[19] Huang P, Nedelcu D, Watanabe M, Jao C, Kim Y, Liu J (2016). Cellular Cholesterol Directly Activates Smoothened in Hedgehog Signaling. Cell.

[20] Rubin LL, de Sauvage FJ (2006). Targeting the Hedgehog pathway in cancer. Nature reviews Drug discovery.

[21] Mill P, Christensen ST, Pedersen LB (2023). Primary cilia as dynamic and diverse signalling hubs in development and disease. Nature reviews Genetics.

[22] Hui CC, Angers S (2011). Gli proteins in development and disease. Annual review of cell and developmental biology.

[23] Kogerman P, Grimm T, Kogerman L, Krause D, Undén AB, Sandstedt B (1999). Mammalian suppressor-of-fused modulates nuclear-cytoplasmic shuttling of Gli-1. Nature cell biology.

[24] Ryan KE, Chiang C (2012). Hedgehog secretion and signal transduction in vertebrates. The Journal of biological chemistry.

[25] Duman-Scheel M, Weng L, Xin S, Du W (2002). Hedgehog regulates cell growth and proliferation by inducing Cyclin D and Cyclin E. Nature.

[26] Regl G, Kasper M, Schnidar H, Eichberger T, Neill GW, Philpott MP (2004). Activation of the BCL2 promoter in response to Hedgehog/GLI signal transduction is predominantly mediated by GLI2. Cancer research.

[27] Velcheti V, Govindan R (2007). Hedgehog signaling pathway and lung cancer. Journal of thoracic oncology: official publication of the International Association for the Study of Lung Cancer.

[28] Chuang PT, McMahon AP (1999). Vertebrate Hedgehog signalling modulated by induction of a Hedgehog-binding protein. Nature.

[29] Hu Z, Liu Y, Tang J, Luo R, Qin J, Mo Z (2023). LncRNA HHIP-AS1 suppresses lung squamous cell carcinoma by stabilizing HHIP mRNA. Life sciences.

[30] Romer J, Curran T (2005). Targeting medulloblastoma: small-molecule inhibitors of the Sonic Hedgehog pathway as potential cancer therapeutics. Cancer research.

[31] Wicking C, Smyth I, Bale A (1999). The hedgehog signalling pathway in tumorigenesis and development. Oncogene.

[32] Raz G, Allen KE, Kingsley C, Cherni I, Arora S, Watanabe A (2012). Hedgehog signaling pathway molecules and ALDH1A1 expression in early-stage non-small cell lung cancer. Lung cancer (Amsterdam, Netherlands).

[33] Watkins DN, Berman DM, Burkholder SG, Wang B, Beachy PA, Baylin SB (2003). Hedgehog signalling within airway epithelial progenitors and in small-cell lung cancer. Nature.

[34] Varnat F, Duquet A, Malerba M, Zbinden M, Mas C, Gervaz P (2009). Human colon cancer epithelial cells harbour active HEDGEHOG-GLI signalling that is essential for tumour growth, recurrence, metastasis and stem cell survival and expansion. EMBO molecular medicine.

[35] Karhadkar SS, Bova GS, Abdallah N, Dhara S, Gardner D, Maitra A (2004). Hedgehog signalling in prostate regeneration, neoplasia and metastasis. Nature.

[36] Kelleher FC (2011). Hedgehog signaling and therapeutics in pancreatic cancer. Carcinogenesis.

[37] Beachy PA, Karhadkar SS, Berman DM (2004). Tissue repair and stem cell renewal in carcinogenesis. Nature.

[38] Schnidar H, Eberl M, Klingler S, Mangelberger D, Kasper M, Hauser-Kronberger C (2009). Epidermal growth factor receptor signaling synergizes with Hedgehog/GLI in oncogenic transformation via activation of the MEK/ERK/JUN pathway. Cancer research.

[39] Riobó NA, Lu K, Ai X, Haines GM, Emerson CP Jr (2006). Phosphoinositide 3-kinase and Akt are essential for Sonic Hedgehog signaling. Proceedings of the National Academy of Sciences of the United States of America.

[40] Luo K (2017). Signaling Cross Talk between TGF-β/Smad and Other Signaling Pathways. Cold Spring Harbor perspectives in biology.

[41] Gialmanidis IP, Bravou V, Amanetopoulou SG, Varakis J, Kourea H, Papadaki H (2009). Overexpression of hedgehog pathway molecules and FOXM1 in non-small cell lung carcinomas. Lung cancer (Amsterdam, Netherlands).

[42] Savani M, Guo Y, Carbone DP, Csiki I (2012). Sonic hedgehog pathway expression in non-small cell lung cancer. Therapeutic advances in medical oncology.

[43] Yuan Z, Goetz JA, Singh S, Ogden SK, Petty WJ, Black CC (2007). Frequent requirement of hedgehog signaling in non-small cell lung carcinoma. Oncogene.

[44] Xu L, Xiong H, Shi W, Zhou F, Zhang M, Hu G (2019). Differential expression of sonic hedgehog in lung adenocarcinoma and lung squamous cell carcinoma. Neoplasma.

[45] Shi I, Hashemi Sadraei N, Duan ZH, Shi T (2011). Aberrant signaling pathways in squamous cell lung carcinoma. Cancer informatics.

[46] Huang L, Walter V, Hayes DN, Onaitis M (2014). Hedgehog-GLI signaling inhibition suppresses tumor growth in squamous lung cancer. Clinical cancer research: an official journal of the American Association for Cancer Research.

[47] Leem YE, Ha HL, Bae JH, Baek KH, Kang JS (2014). CDO, an Hh-coreceptor, mediates lung cancer cell proliferation and tumorigenicity through Hedgehog signaling. PloS one.

[48] Bermudez O, Hennen E, Koch I, Lindner M, Eickelberg O (2013). Gli1 mediates lung cancer cell proliferation and Sonic Hedgehog-dependent mesenchymal cell activation. PloS one.

[49] Rowbotham SP, Goruganthu MUL, Arasada RR, Wang WZ, Carbone DP, Kim CF (2022). Lung Cancer Stem Cells and Their Clinical Implications. Cold Spring Harbor perspectives in medicine.

[50] Wang H, Si S, Jiang M, Chen L, Huang K, Yu W (2021). Leukemia inhibitory factor is involved in the pathogenesis of NSCLC through activation of the STAT3 signaling pathway. Oncology letters.

[51] Apte RS, Chen DS, Ferrara N (2019). VEGF in Signaling and Disease: Beyond Discovery and Development. Cell.

[52] Brentnall TA, Lai LA, Coleman J, Bronner MP, Pan S, Chen R (2012). Arousal of cancer-associated stroma: overexpression of palladin activates fibroblasts to promote tumor invasion. PloS one.

[53] Mondal S, Adhikari N, Banerjee S, Amin SA, Jha T (2020). Matrix metalloproteinase-9 (MMP-9) and its inhibitors in cancer: A minireview. European journal of medicinal chemistry.

[54] Yoo YA, Kang MH, Lee HJ, Kim BH, Park JK, Kim HK (2011). Sonic hedgehog pathway promotes metastasis and lymphangiogenesis via activation of Akt, EMT, and MMP-9 pathway in gastric cancer. Cancer research.

[55] Zhang Q, Wang L, Wang S, Cheng H, Xu L, Pei G (2022). Signaling pathways and targeted therapy for myocardial infarction. Signal transduction and targeted therapy.

[56] Lin CC, Kuo IY, Wu LT, Kuan WH, Liao SY, Jen J (2020). Dysregulated Kras/YY1/ZNF322A/Shh transcriptional axis enhances neo-angiogenesis to promote lung cancer progression. Theranostics.

[57] Lei X, Zhong Y, Huang L, Li S, Fu J, Zhang L (2020). Identification of a novel tumor angiogenesis inhibitor targeting Shh/Gli1 signaling pathway in Non-small cell lung cancer. Cell death & disease.

[58] Kasiri S, Chen B, Wilson AN, Reczek A, Mazambani S, Gadhvi J (2020). Stromal Hedgehog pathway activation by IHH suppresses lung adenocarcinoma growth and metastasis by limiting reactive oxygen species. Oncogene.

[59] Skobe M, Hawighorst T, Jackson DG, Prevo R, Janes L, Velasco P (2001). Induction of tumor lymphangiogenesis by VEGF-C promotes breast cancer metastasis. Nature medicine.

[60] Hwang J, Kang MH, Yoo YA, Quan YH, Kim HK, Oh SC (2014). The effects of sonic hedgehog signaling pathway components on non-small-cell lung cancer progression and clinical outcome. World journal of surgical oncology.

[61] Takebe N, Harris PJ, Warren RQ, Ivy SP (2011). Targeting cancer stem cells by inhibiting Wnt, Notch, and Hedgehog pathways. Nature reviews Clinical oncology.

[62] Yang X, Yu L, Shao M, Yang H, Qi K, He G (2025). N6-methyladenosine-modified GPX2 impacts cancer cell stemness and TKI resistance through regulating of redox metabolism. Cell death & disease.

[63] Moustafa EM, Abdel Salam HS, Mansour SZ (2022). Withania somnifera Modulates Radiation-Induced Generation of Lung Cancer Stem Cells via Restraining the Hedgehog Signaling Factors. Dose-response: a publication of International Hormesis Society.

[64] Singh S, Trevino J, Bora-Singhal N, Coppola D, Haura E, Altiok S (2012). EGFR/Src/Akt signaling modulates Sox2 expression and self-renewal of stem-like side-population cells in non-small cell lung cancer. Molecular cancer.

[65] Takahashi K, Yamanaka S (2006). Induction of pluripotent stem cells from mouse embryonic and adult fibroblast cultures by defined factors. Cell.

[66] Chang KJ, Yin JZ, Huang H, Li B, Yang MH (2020). Arsenic trioxide inhibits the growth of cancer stem cells derived from small cell lung cancer by downregulating stem cell-maintenance factors and inducing apoptosis via the Hedgehog signaling blockade. Translational lung cancer research.

[67] Dong H, Zeng L, Chen W, Zhang Q, Wang F, Wu Y (2023). N6-methyladenine-mediated aberrant activation of the lncRNA SOX2OT-GLI1 loop promotes non-small-cell lung cancer stemness. Cell death discovery.

[68] Wang F, Sun Y, Huang X, Qiao C, Zhang W, Liu P (2021). Sulforaphane inhibits self-renewal of lung cancer stem cells through the modulation of sonic Hedgehog signaling pathway and polyhomeotic homolog 3. AMB Express.

[69] Choi HS, Kim SL, Kim JH, Lee DS (2020). The FDA-Approved Anti-Asthma Medicine Ciclesonide Inhibits Lung Cancer Stem Cells through Hedgehog Signaling-Mediated SOX2 Regulation. International journal of molecular sciences.

[70] Wu HJ, Chu PY (2019). Role of Cancer Stem Cells in Cholangiocarcinoma and Therapeutic Implications. International journal of molecular sciences.

[71] Wang X, Eichhorn PJA, Thiery JP (2023). TGF-β, EMT, and resistance to anti-cancer treatment. Seminars in cancer biology.

[72] Messaritakis I, Kotsakis A, Georgoulias V (2017). Association of epithelial-to-mesenchymal transition circulating tumor cells in non-small cell lung cancer (NSCLC) molecular subgroups. Journal of thoracic disease.

[73] Ahmad A, Maitah MY, Ginnebaugh KR, Li Y, Bao B, Gadgeel SM (2013). Inhibition of Hedgehog signaling sensitizes NSCLC cells to standard therapies through modulation of EMT-regulating miRNAs. Journal of hematology & oncology.

[74] Das S, Samant RS, Shevde LA (2013). Nonclassical activation of Hedgehog signaling enhances multidrug resistance and makes cancer cells refractory to Smoothened-targeting Hedgehog inhibition. The Journal of biological chemistry.

[75] Jiang L, Huang J, Hu Y, Lu P, Luo Q, Wang L (2020). Gli promotes tumor progression through regulating epithelial-mesenchymal transition in non-small-cell lung cancer. Journal of cardiothoracic surgery.

[76] Dong Z, Wang Y, Ding V, Yan X, Lv Y, Zhong M (2020). GLI1 activation is a key mechanism of erlotinib resistance in human non-small cell lung cancer. Oncol Lett.

[77] Gottesman MM, Fojo T, Bates SE (2002). Multidrug resistance in cancer: role of ATP-dependent transporters. Nature reviews Cancer.

[78] Zhang Y, Vagiannis D, Budagaga Y, Sabet Z, Hanke I, Rozkoš T (2022). Sonidegib potentiates the cancer cells' sensitivity to cytostatic agents by functional inhibition of ABCB1 and ABCG2 in vitro and ex vivo. Biochemical pharmacology.

[79] Del Re M, Arrigoni E, Restante G, Passaro A, Rofi E, Crucitta S (2018). Concise Review: Resistance to Tyrosine Kinase Inhibitors in Non-Small Cell Lung Cancer: The Role of Cancer Stem Cells. Stem cells (Dayton, Ohio).

[80] Huang A, Cheng J, Zhan Y, Zhou F, Xuan Y, Wang Y (2024). Hedgehog ligand and receptor cooperatively regulate EGFR stability and activity in non-small cell lung cancer. Cellular oncology (Dordrecht, Netherlands).

[81] Zheng H, Tang Y, Zang H, Luo J, Zhou H, Zhan Y (2025). Itraconazole Reversing Acquired Resistance to Osimertinib in NSCLC by Inhibiting the SHH/DUSP13B/p-STAT3 Axis. Advanced science (Weinheim, Baden-Wurttemberg, Germany).

[82] Seidl C, Panzitt K, Bertsch A, Brcic L, Schein S, Mack M (2020). MicroRNA-182-5p regulates hedgehog signaling pathway and chemosensitivity of cisplatin-resistant lung adenocarcinoma cells via targeting GLI2. Cancer letters.

[83] Tu YC, Yeh WC, Yu HH, Lee YC, Su BC (2022). Hedgehog Suppresses Paclitaxel Sensitivity by Regulating Akt-Mediated Phosphorylation of Bax in EGFR Wild-Type Non-Small Cell Lung Cancer Cells. Frontiers in pharmacology.

[84] Sims-Mourtada J, Izzo JG, Ajani J, Chao KS (2007). Sonic Hedgehog promotes multiple drug resistance by regulation of drug transport. Oncogene.

[85] Li LB, Yang LX, Liu L, Liu FR, Li AH, Zhu YL (2024). Targeted inhibition of the HNF1A/SHH axis by triptolide overcomes paclitaxel resistance in non-small cell lung cancer. Acta pharmacologica Sinica.

[86] Lee WK, Frank T (2021). Teaching an old dog new tricks: reactivated developmental signaling pathways regulate ABCB1 and chemoresistance in cancer. Cancer drug resistance (Alhambra, Calif).

[87] Wen Y, Li K, Ni M, Jiang H, Wu H, Yu Q (2024). Dendritic Polylysine with Paclitaxel and Triptolide Codelivery for Enhanced Cancer Ferroptosis through the Accumulation of ROS. ACS applied materials & interfaces.

[88] Tsai YS, Woodcock MG, Azam SH, Thorne LB, Kanchi KL, Parker JS (2022). Rapid idiosyncratic mechanisms of clinical resistance to KRAS G12C inhibition. The Journal of clinical investigation.

[89] Lauth M, Bergström A, Shimokawa T, Tostar U, Jin Q, Fendrich V (2010). DYRK1B-dependent autocrine-to-paracrine shift of Hedgehog signaling by mutant RAS. Nature structural & molecular biology.

[90] Lee C, Yi J, Park J, Ahn B, Won YW, Jeon J (2024). Hedgehog signalling is involved in acquired resistance to KRAS(G12C) inhibitors in lung cancer cells. Cell death & disease.

[91] Pugacheva EN, Jablonski SA, Hartman TR, Henske EP, Golemis EA (2007). HEF1-dependent Aurora A activation induces disassembly of the primary cilium. Cell.

[92] Rohatgi R, Milenkovic L, Scott MP (2007). Patched1 regulates hedgehog signaling at the primary cilium. Science (New York, NY).

[93] Katoh M (2019). Genomic testing, tumor microenvironment and targeted therapy of Hedgehog-related human cancers. Clinical science (London, England: 1979).

[94] Hanna A, Metge BJ, Bailey SK, Chen D, Chandrashekar DS, Varambally S (2019). Inhibition of Hedgehog signaling reprograms the dysfunctional immune microenvironment in breast cancer. Oncoimmunology.

[95] Grund-Gröschke S, Ortner D, Szenes-Nagy AB, Zaborsky N, Weiss R, Neureiter D (2020). Epidermal activation of Hedgehog signaling establishes an immunosuppressive microenvironment in basal cell carcinoma by modulating skin immunity. Molecular oncology.

[96] Petty AJ, Li A, Wang X, Dai R, Heyman B, Hsu D (2019). Hedgehog signaling promotes tumor-associated macrophage polarization to suppress intratumoral CD8+ T cell recruitment. The Journal of clinical investigation.

[97] Zhu R, Huang J, Qian F (2025). The role of tumor-associated macrophages in lung cancer. Frontiers in immunology.

[98] Petty AJ, Dai R, Lapalombella R, Baiocchi RA, Benson DM, Li Z (2021). Hedgehog-induced PD-L1 on tumor-associated macrophages is critical for suppression of tumor-infiltrating CD8+ T cell function. JCI insight.

[99] Mehlman C, Takam Kamga P, Costantini A, Julié C, Dumenil C, Dumoulin J (2021). Baseline Hedgehog Pathway Activation and Increase of Plasma Wnt1 Protein Are Associated with Resistance to Immune Checkpoint Inhibitors in Advanced Non-Small-Cell Lung Cancer. Cancers.

[100] Qiu J, Li X, He Y, Wang Q, Li J, Wu J (2022). Identification of comutation in signaling pathways to predict the clinical outcomes of immunotherapy. Journal of translational medicine.

[101] Lim S, Lim SM, Kim MJ, Park SY, Kim JH (2019). Sonic Hedgehog Pathway as the Prognostic Marker in Patients with Extensive Stage Small Cell Lung Cancer. Yonsei medical journal.

[102] Stratigos AJ, Sekulic A, Peris K, Bechter O, Prey S, Kaatz M (2021). Cemiplimab in locally advanced basal cell carcinoma after hedgehog inhibitor therapy: an open-label, multi-centre, single-arm, phase 2 trial. The Lancet Oncology.

[103] Doyle CM, Cevik J, Ramakrishnan A (2025). Safety and Efficacy of Vismodegib and Sonidegib in Advanced Basal Cell Carcinoma of the Head and Neck: A Systematic Review and Meta-Analysis. Head & neck.

[104] Awad AA, Shaban AY, Mohammed F, Marey MM, Aldemerdash MA, Abbas AW (2025). Glasdegib combined with chemotherapy in the treatment of patients with acute myeloid leukemia: a comprehensive meta-analysis. Investigational new drugs.

[105] Fan J, Zhang X, Wang S, Chen W, Li Y, Zeng X (2019). Regulating autophagy facilitated therapeutic efficacy of the sonic Hedgehog pathway inhibition on lung adenocarcinoma through GLI2 suppression and ROS production. Cell death & disease.

[106] Gerber DE, Putnam WC, Fattah FJ, Kernstine KH, Brekken RA, Pedrosa I (2020). Concentration-dependent Early Antivascular and Antitumor Effects of Itraconazole in Non-Small Cell Lung Cancer. Clinical cancer research: an official journal of the American Association for Cancer Research.

[107] Mohamed AW, Elbassiouny M, Elkhodary DA, Shawki MA, Saad AS (2021). The effect of itraconazole on the clinical outcomes of patients with advanced non-small cell lung cancer receiving platinum-based chemotherapy: a randomized controlled study. Medical oncology (Northwood, London, England).

